# The visuomotor synchronization immersive virtual reality of a depression avatar in a stigma context experience mobilizes the fronto-parietal cortex and anterior insula

**DOI:** 10.3389/fnbeh.2025.1526684

**Published:** 2025-01-31

**Authors:** Kelssy Hitomi dos Santos Kawata, Wey Guan Lem, Koki Ono, Hiroshi Oyama

**Affiliations:** ^1^Graduate School of Medicine, The University of Tokyo, Tokyo, Japan; ^2^Graduate School of Interdisciplinary Information Studies, The University of Tokyo, Tokyo, Japan

**Keywords:** embodiment, avatar, stigma, fMRI, immersive virtual reality, illusion, visuomotor synchrony, depression

## Abstract

**Introduction:**

The gradual synchronization of the movement of one’s real hand with a virtual one can effectively induce a sense of embodiment (SoE) with an avatar with depression. Although neuroimaging studies have explored the neural correlates of some SoE subcomponents of visuomotor synchronization, the neural correlates of individual differences in SoE and how humans acquire virtual body representations through SoE subcomponents remain to be investigated.

**Methods:**

Here, we used the right hand of a virtual patient with depression in immersive virtual reality (IVR) to induce SoE in participants and measured whole brain activity using functional magnetic resonance imaging (fMRI). Participants were instructed to listen to the audio recording of the IVR experience and visualize movements during the fMRI scan. fMRI data were acquired before and immediately after the visuomotor synchronization IVR experience (target condition) or an asynchronized video experience (control condition), followed by embodiment measures related to the two types of experiences.

**Results:**

All five subcomponents of SoE (sense of ownership, sense of agency, sense of localization, appearance, and response to stimuli) were significantly increased during the visuomotor synchronization IVR experience compared with the asynchronized video experience. A significant negative effect of the SoE score was identified in the frontoparietal and anterior insula only for the visuomotor synchronization IVR experience of guiding the virtual right hand of the avatar with depression, implicating interoceptive and multisensory integration.

**Discussion:**

We demonstrated that all five subcomponents of the SoE were present, and that decreased activity in the frontoparietal and anterior insula were crucial brain regions for the virtual human body to be perceived as one’s own body and promote conscious feelings of embodiment.

## 1 Introduction

Public stigma is a misconception held by the general population toward socially disadvantaged groups, such as individuals with depression. A first-person perspective (1PP) using an avatar with depression in an immersive virtual reality (IVR) reproducing the feeling of stigma has been suggested as a way to change this misconception and reduce the stigma of depression through the sense of embodiment (SoE) (Lem et al., 2024). In general, the SoE in 1PP IVR using an avatar is caused by a gradual perceptual acceptance of the illusion of the avatar, which depends on visuomotor synchronization and the combination of multisensory information (Sanchez-Vives et al., 2010; Kilteni et al., 2012; Apps and Tsakiris, 2014; Johnston et al., 2023). The SoE emerges from three main underlying subcomponents: sense of ownership (SoO) (“The virtual body is my real body”), sense of agency (SoA) (“I can exercise control over the virtual body”), and sense of localization (SoL) (“My body is located in the virtual body”). Furthermore, although controversial, previous studies have shown that the detailed appearance of the virtual embodied avatar might act as an additional component in the construction of the SoO (Waltemate et al., 2018; Pyasik et al., 2020), while others have found contradictory results (Slater et al., 2010; Kilteni et al., 2013; Peck et al., 2013; Banakou et al., 2016).

Several studies have demonstrated that when a person in the general population is virtually embodied in a stigmatized avatar through synchronized visuomotor movements of the entire body (Kilteni et al., 2013; Peck et al., 2013; Banakou et al., 2016; Banakou et al., 2018) or part of the body (de Borst et al., 2020; Lem et al., 2024), their perceptions, attitudes, and behaviors toward persons in the stigmatized group change positively. For example, a visuomotor synchrony experience using hand tracking on a virtual avatar with depression showed an increase in SoE and a reduction in social distance (i.e., stigma) relative to individuals with depression (Lem et al., 2024). Regarding the SoE subcomponents, the use of visuomotor synchronization of the full body of an older virtual avatar and a Black virtual avatar induced the illusion of SoO and SoA, consequently reducing implicit bias and improving identification with older individuals (Banakou et al., 2018) and Black individuals (Kilteni et al., 2013; Peck et al., 2013; Banakou et al., 2016). Similarly, regarding the visuomotor synchrony of a virtual body part (i.e., head), a previous study also found higher SoO and improved self-identification with a virtual female avatar in the context of domestic violence (de Borst et al., 2020). Although some studies have shown how SoE subcomponents behave in 1PP IVR experiences of stigma (Kilteni et al., 2013; Peck et al., 2013; Banakou et al., 2016; Banakou et al., 2018; de Borst et al., 2020), to date, how humans acquire virtual body representation through SoE subcomponents using a standardized embodiment questionnaire on the stigma of depression, or even stigma in general, has not been evaluated. Furthermore, although there is considerable individual variation in how people feel embodied toward a virtual avatar (Gonzalez-Franco and Peck, 2018), the neural correlates of individual differences in SoE using the 1PP IVR experience of stigma in an individual with depression are unknown.

Although the use of the 1PP IVR stigma experience has been a promising application for changing human behavior toward a stigmatized group, the neural correlates underlying SoE obtained by visuomotor synchronization are far from being fully understood. Some have proposed that the frontoparietal and anterior insula (AIN) are involved in developing SoE from visuo-tactile synchronization in real-world environments (Serino et al., 2013; Blanke et al., 2015; Guterstam et al., 2015; Park and Blanke, 2019; Owens, 2023). Specifically, acquiring SoE using visuo-tactile synchronization leads to activation of the middle frontal gyrus (MFG) and inferior frontal gyrus (IFG), similar to the induction that occurs in the rubber hand illusion (RHI) (Chae et al., 2015) and the body-swapping illusion (Petkova et al., 2011). Furthermore, the AIN is involved in interoceptive processes in the RHI (Guterstam et al., 2013; Limanowski et al., 2014; Chae et al., 2015) and the body-swapping illusion (Petkova et al., 2011; Serino et al., 2013) through visuo-tactile synchronization. Regarding the parietal lobe, several brain regions in the posterior parietal cortex (PPC), such as the superior parietal lobule (SPL), angular gyrus (AnG), and intraparietal sulcus (IPS), were related to multisensory integration during visuo-tactile synchronization (Petkova et al., 2011; Guterstam et al., 2013; Grivaz et al., 2017). Multisensory integration in the PPC is related to the transformation of visual, tactile, and proprioceptive signals into stable spatial representations of body parts that use different sensory (i.e., receiving external and internal stimuli) and perceptual (i.e., interpreting the stimuli) modalities (Serino et al., 2013).

Evidence suggests that there is a core set of brain regions crucial for the virtual avatar to be perceived as one’s own body, regardless of the type of synchronization. The frontoparietal and AIN brain regions focus on illusion induction, interoceptive processes, and multisensory integration processing related to visuo-tactile synchronization manipulations, as described in the previous paragraph. These brain regions are also activated when participants experience the SoA during visuomotor synchronization in real-world and IVR environments (Uhlmann et al., 2020; Cai et al., 2024) and when participants experience fear during the SoE of a virtual avatar, with activation of the MFG, IFG, and IPS; this fear threat is generally signaled by the activation of the AIN in 3D videos of virtual reality (VR) and in IVR environments (Seinfeld et al., 2021; de Borst and de Gelder, 2022).

In terms of individual variations in visuomotor synchronization, neuroimaging studies have explored the neural correlates of some subcomponents and processes related to the SoE by correlational analysis using the sense of presence score (Clemente et al., 2014), temporal precision judgment time (Cai et al., 2024), and face and body stimuli tasks (Seinfeld et al., 2021). Similar to previous studies, these studies highlighted similar frontoparietal and AIN brain regions. They compared groups with different levels of neural activation during the manipulation of 3D video and IVR environment exposure-related experiences with visuomotor synchronization effects, such as moving a joystick freely, visualizing an avatar’s hand in motor movement synchrony with a real hand, and embodying the avatar of an abuse victim in visuomotor synchrony (Clemente et al., 2014; Seinfeld et al., 2021; Cai et al., 2024). For the MFG, the greater the sense of presence the participants had toward the 3D videos of the VR environment, the lower their neural activation in the MFG (Clemente et al., 2014). The SoA in controlling an avatar was negatively associated with increased activity in the IFG and AnG (Cai et al., 2024). Moreover, decreased activation of the AIN has been associated with embodiment of victims of virtual abuse (Seinfeld et al., 2021). However, to our knowledge, it is unknown whether the processes described above can be extended to individual differences in the SoE during visuomotor synchronization with an avatar with depression in 1PP IVR.

In the present study, we investigated how humans acquire virtual body representations through SoE subcomponents and the neural correlates of individual differences in SoE using visuomotor synchronization with an avatar with depression experiencing stigma in IVR. The participants were exposed to both visuomotor synchrony IVR and asynchrony video experiences, with videos selected for their established roles in asynchrony studies (Gall et al., 2021; Diel et al., 2023; Lem et al., 2024). In the visuomotor synchronization IVR, participants experienced visuomotor synchronization with an avatar with depression, which included a virtual office environment where the participants took on the role of a company employee with mild symptoms of depression (American Psychiatric Association, 2013) such as inability to concentrate at work, loss of interest in previously interesting activities (anhedonia), sleep deprivation (insomnia), loss of appetite, tiredness, and inability to move (lethargic) in the 1PP and experienced stigma from two coworkers (e.g., receiving a document that was rejected by the boss through grabbing a paper), as well as support from a colleague and his/her mother. The video content used in the video experience was the same as that in the visuomotor synchrony IVR experience but without immersion and visuomotor synchronization. Additionally, each participant listened to an auditory fMRI task (non-visual auditory content of the experience) before and after the visuomotor synchronization IVR and asynchrony video experiences, followed by embodiment measure and perception of re-experience measure related to the two types of experiences (Gonzalez-Franco and Peck, 2018; Arai et al., 2022; Lem et al., 2024).

We hypothesized that the SoE subcomponents (i.e., SoO, SoA, SoL, appearance, and response to stimuli) would increase in the visuomotor synchronization IVR experience that uses multisensory information (i.e., vision, motor, proprioception, hand location, and appearance) and decrease in the asynchronized video experience. Based on RHI and body swap illusion studies (Petkova et al., 2011; Guterstam et al., 2013; Limanowski et al., 2014; Blanke et al., 2015; Chae et al., 2015; Guterstam et al., 2015; Owens, 2023), we postulated that similar brain activation, comprising the MFG, IFG, AIN, and PPC, would be activated as interoceptive and multisensory integration, which would lead to an SoE toward the hand of a virtual avatar with depression during visuomotor synchronization IVR. Finally, considering the literature on visuomotor synchronization of 3D video and IVR environment exposures, we speculated that the MFG, IFG, AIN, and PPC would show decreased activation during the increase in SoE compared to the asynchronized video experience.

## 2 Materials and methods

### 2.1 Participants

Forty healthy adults assessed for eligibility, 36 (13 female; age [mean ± *SD*] = 24.00 ± 6.1 years, age range = 19.0–46.0 years) were recruited from undergraduate and graduate schools of The University of Tokyo through social media advertisements between November 2022 and February 2023. The inclusion criteria consisted of being native Japanese citizens and right-handed with normal or corrected-to-normal vision and auditory processes. In addition, none of the participants reported any neurological or neuropsychiatric disorders or psychotropic medication use. Written informed consent was obtained from all participants. The experiment was conducted in accordance with the Declaration of Helsinki, and all procedures were approved by the Research Ethics Committee of the Faculty of Medicine and Graduate School of Medicine of the University of Tokyo (2019099NI).

### 2.2 Procedures

#### 2.2.1 Experimental procedure overview

Each participant performed three consecutive phases of the experiment (i.e., an fMRI baseline, visuomotor synchrony with an IVR depression avatar or asynchrony with a video depression avatar, and fMRI post-experience). In the fMRI baseline and post-experiences, the participants performed the auditory version of the depression avatar experience (i.e., depression avatar auditory experience) in a magnetic resonance imaging (MRI) scanner. After the fMRI baseline experience, the participants were given approximately 15 min of rest before performing the visuomotor synchrony in an IVR or asynchrony with video experiences. During the visuomotor synchrony IVR/target experience, participants were seated on a chair with 360-degree rotation, where the experience was visuomotor synchrony of an avatar with depression in IVR presented via a head-mounted display (HMD). The experimenter briefed the participants on the use of the HMD. After setting up the HMD, the participants set the active noise cancelation intra-auricular earphones and underwent a visuomotor synchrony IVR experience. The experience began when the participants placed their hands a few centimeters in front of the midline of their body, at position [0, 0, 0] on the XYZ plane of the virtual environment, for the HMD to detect their hands. At the end of the visuomotor synchrony IVR experience, the participants were instructed to close their eyes before removing the HMD and were blindfolded after removing the HMD.

The asynchrony of the video/control experience was shown on a laptop screen, and the participants were seated on a chair with 360-degree rotation with active noise cancelation intra-auricular earphones. The participants were instructed to start an asynchronous video version of the IVR depression avatar experience using a computer mouse. The experience began when participants clicked on the “play” button on the video computer screen.

After both types of experience, the blindfolded participants were guided to the MRI scanning room by the experimenter for the fMRI post-experience. Similar to the baseline fMRI experience, the participants used a sleeping mask over their eyes and were instructed to listen to the contents of the depression avatar auditory experience stimulus during fMRI scanning and imagine themselves in the situation. Structural brain images were acquired during the post-experience asynchronous video. The participants were asked to evaluate their subjective SoE during the visuomotor synchrony IVR or asynchrony video experiences during the post-experience fMRI.

#### 2.2.2 Experimental design

**Figure 1:**
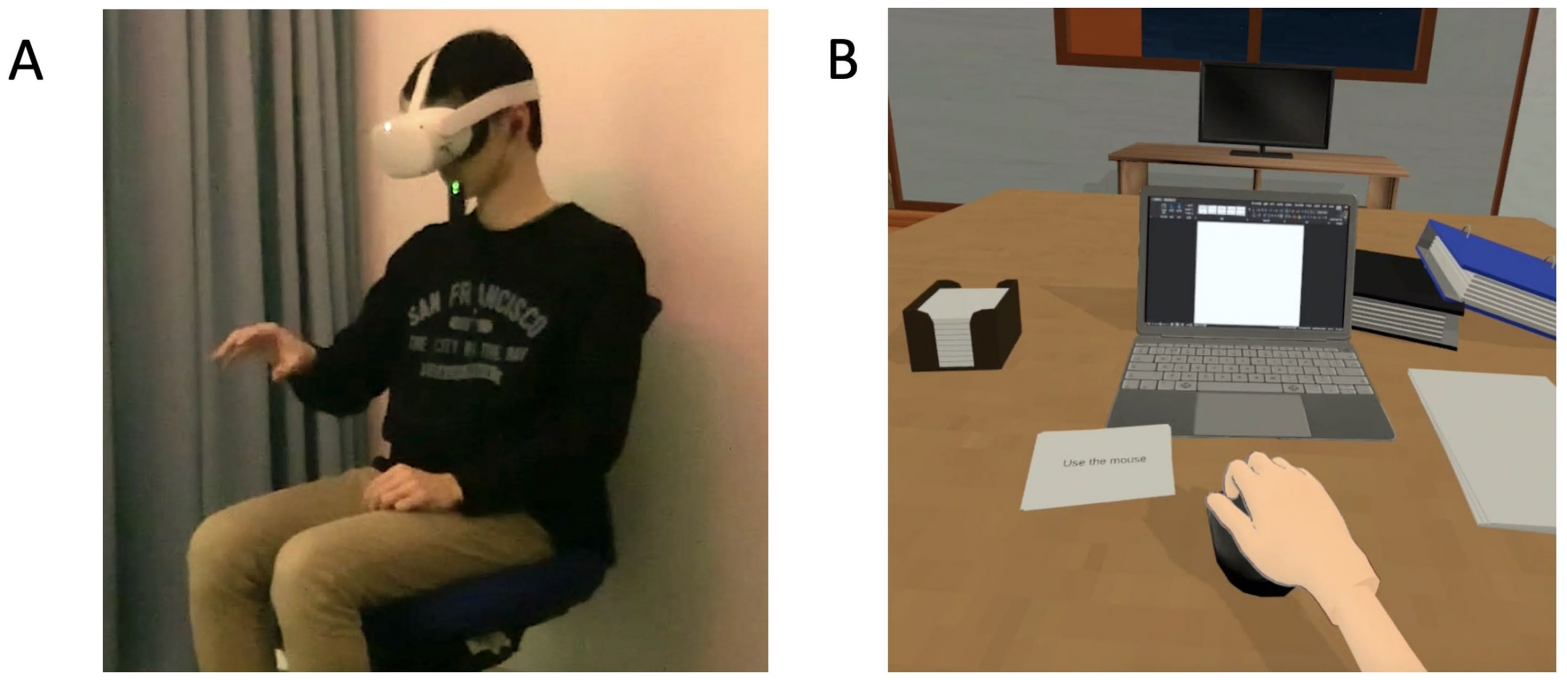
**(A)** Depiction of the setup used for the visuomotor synchrony with an avatar with depression in the context of stigma in IVR. **(B)** Screenshot of the depression avatar in the context of stigma in the visuomotor synchrony IVR and asynchrony video experiences. IVR: immersive virtual reality.

**Figure 2:**
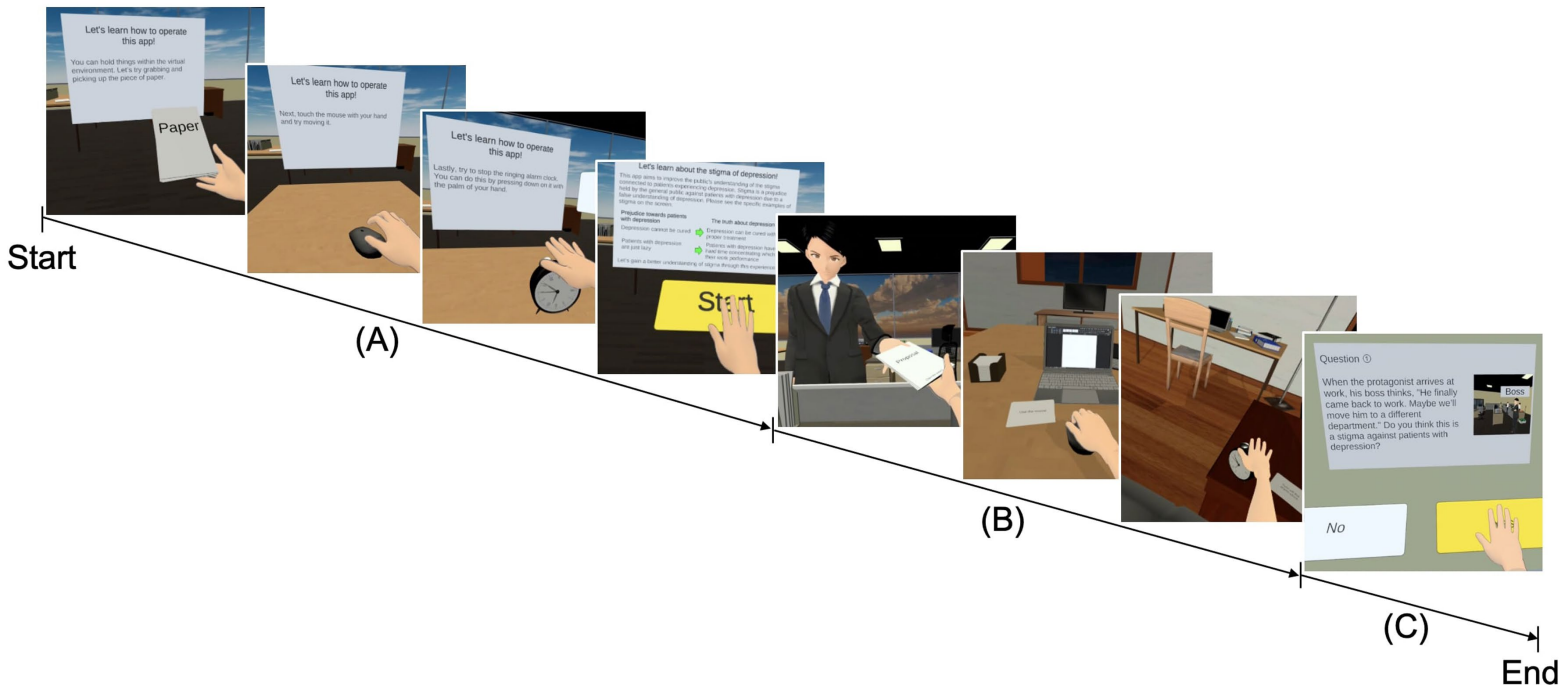
Progression screenshot of the visuomotor synchrony with an avatar with depression in the context of stigma in IVR. **(A)** Tutorial phase: the participant was familiarized with the visuomotor synchronized activities that will be carried out in the main scenario (i.e., picking up the paper, moving the mouse, turning off the alarm clock, and pressing some functional buttons), and a brief description of the stigma of depression was also provided. **(B)** Main scenario: the participant assumed the role of a company employee with mild symptoms of depression and experienced the stigma of depression through visuomotor synchrony with the avatar with depression. **(C)** Quiz: the participant answered five questions related to the application experienced through a visuomotor synchrony IVR button. IVR, immersive virtual reality.

This was a crossover randomized controlled trial (RCT). Participants were randomly assigned to start with one of the two types of experience with the same content: (1) a visuomotor synchrony IVR experience using an HMD (Figure 1) or (2) an asynchrony video experience presented on a computer screen. All participants were required to participate in both days of the study. Participants underwent visuomotor synchrony of an IVR depression avatar experience on the first measurement day (Day 1). After a washout period of approximately 1–49 days, they underwent asynchrony of a video depression avatar experience on the second measurement day (Day 2), and vice versa. This study adhered to the CONSORT guidelines (Dwan et al., 2019) and was registered in the University Hospital Medical Information Network Clinical Trials Registry (UMIN-CTR) (Identifier: UMIN000043020, First registration date: 20/01/2021), Japan.

#### 2.2.3 Experimental equipment

The visuomotor synchrony of a IVR depression avatar experience was presented via an HMD (“Oculus Quest 2”; 120 Hz refresh rate, 1832 × 1920 resolution per eye, 104° field of view horizontal) with active noise cancelation intra-auricular earphones (Bose QC 20, United States), and tracking of the right and left hands was used as a means of synchronizing with objects and functions within the virtual environment. The IVR was developed using the Unity3D game engine (Unity Technologies, San Francisco, CA, United States) and computer-aided design (CAD) software Blender. C# was selected as the programming language. VRoid Studio v1.11.1, a hub of freely available 3D anime-based models, was used to create the virtual characters.

Asynchrony with the video depression avatar was experienced by viewing a laptop screen (Windows OS 10, refresh rate of 60 Hz, 1920 × 1,080 non-interlaced monitor resolution) with active noise cancelation intra-auricular earphones (Bose QC 20, United States). The laptop screen was positioned below the participant’s eye level, and the distance from the screen to the participant’s eyes was approximately 45 cm (a visual angle of ~42°). The asynchrony video experience was started when participants clicked on the “play” button in Windows Media Player using the computer mouse. The content of the asynchrony video was the same as the visuomotor synchrony IVR experience; however, no immersive visual-motor synchronization (i.e., the depression avatar moved independently) was required of the participants while watching the video.

#### 2.2.4 Visuomotor synchrony of the stigma experience of an avatar with depression in IVR

The visuomotor synchrony IVR experiment was adapted from our previous studies (Lem et al., 2022; Lem et al., 2024), in which participants viewed a virtual representation of their right hand in the same position as their actual hand. The visuomotor synchrony IVR experience content consisted of three phases: tutorial, main scenario, and reflection. In the tutorial phase, the participant was familiarized with the visual-motor activities to be carried out in the main scenario (i.e., grabbing a paper, holding a computer mouse, pressing the snooze button on an alarm clock, and pressing some functional buttons such as “start” and “next”), and a brief description of the stigma was provided before proceeding (Figure 2A). In the main scenario phase, the participant assumed the role of a company employee with mild symptoms of depression (American Psychiatric Association, 2013) in the 1PP, experienced stigma from two coworkers, and was supported by a colleague and his mother (Figure 2B). In terms of mild symptoms of depression, the participants were instructed to experience symptoms portrayed in IVR, such as inability to concentrate at work, anhedonia, insomnia, loss of appetite, tiredness, and lethargic. In this context, in terms of the visuomotor synchrony experience, the participants were instructed to receive a document that was rejected by the boss (i.e., grabbing a paper), do extra work at home with the computer (i.e., holding a computer mouse), and turn off the alarm clock owing to the lack of interest in going to work (i.e., pressing the snooze button on the alarm clock) using the right hand. For the reflection phase, the participants completed five questions related to the main scenario to confirm their understanding of public stigma using their right hand (Figure 2C).

### 2.3 Depression avatar auditory fMRI task

In addition to the visuomotor synchrony IVR avatar experience, each participant performed an auditory task as the depression avatar during fMRI to assess the association between the differences in brain activation at baseline and after the visuomotor synchrony IVR and asynchrony experiences to measure the SoE. The instructions and the depression avatar auditory fMRI task (Figure 2B) without visual information were presented using MRI-compatible headphones (SS3300; Avotec, Inc., Stuart, FL, United States). The instructions for performing the task were presented aurally for 6 s in each block, during which the participants were asked to listen to the audio stimulus and imagine themselves in the situation. The depression avatar auditory fMRI task was sounded for 240 s and contained the same auditory information presented in the main scenario phase of the visuomotor synchrony IVR or asynchrony video experience (Figure 2B), followed by a rest of 15 s. Four blocks were performed over 19 min for each baseline and post experience. The depression avatar auditory fMRI task stimulus was presented using PsychoPy v2021.2.3 (Peirce, 2007, 2008).

### 2.4 Assessments

#### 2.4.1 Embodiment measure

The evaluation of embodiment for each type of experience was assessed using a 14-item embodiment questionnaire rated on a 7-Likert scale (−3 = *strongly disagree* to 3 = *strongly agree*). The questionnaire was adopted from previous studies on the embodiment of virtual avatars (Gonzalez-Franco and Peck, 2018; Arai et al., 2022), and it asked whether a distinction was made between the participant’s hands and the virtual hands. The total embodiment (i.e., SoE) and five subdomain scores (i.e., SoO, SoA, SoL, appearance, and response to external stimuli) were calculated based on the original study (Gonzalez-Franco and Peck, 2018), excluding questions not assessed in this study. In our dataset, Cronbach’s alpha was 0.81 for the visuomotor synchrony IVR and 0.85 for the asynchrony video experiences to acquire SoE.

#### 2.4.2 Perception of re-experience measure

Participants were asked to respond intuitively to how they were relieved of the visuomotor synchrony of an IVR and the asynchrony of a video depression avatar experience inside the MRI scanner. The participants responded to 10 questions rated on a 7-point Likert scale (1 = strongly disagree to 7 = strongly agree) based on our previous study (Lem et al., 2024). All questions and total perception of the re-experience scores were considered.

### 2.5 MRI data acquisition

All fMRI data were acquired using a 3-T whole-body MRI system (MAGNETOM Prisma; Siemens, Erlangen, Germany) with a 64-channel head coil. In the depression avatar auditory fMRI task, we acquired 39 continuous slices of functional images per volume (field of view [FOV] = 192 × 192 mm^2^, 64 × 64 matrix, slice thickness = 3 mm, slice gap = 0.75 mm) using an echo planar imaging (EPI) pulse sequence (repetition time [TR] = 2,000 ms, echo time [TE] = 25 ms, and flip angle [FA] = 90°). We acquired 570 volumes at baseline and post-experience. All the slices were tilted 30° from the anterior/posterior commissure plane to the forehead. We also acquired whole-brain high-resolution T1-weighted images (1 × 1 × 1 mm^3^) with a magnetization-prepared rapid acquisition gradient echo sequence (TR = 2,000 ms, TE = 2.9 ms, FA = 9°; FOV = 256 × 256 × 256 mm^3^). We visually inspected all structural and functional images to assess image quality and movement artifacts. In addition, all participants were instructed to relax and avoid falling asleep.

### 2.6 Analysis

#### 2.6.1 Behavioral data analysis

We compared demographic characteristics between groups by order of the experiences (visuomotor synchrony IVR then asynchrony video vs. asynchrony video then visuomotor synchrony IVR) using t-tests for continuous variables and chi-square tests for categorical variables. The Shapiro–Wilk test was used to confirm the normality of the embodiment measure at post-experience (embodiment measure: visuomotor synchrony IVR, *p* = 0.64; asynchrony video, *p* = 0.52). The Shapiro–Wilk test showed a normal distribution for the embodiment measure. A paired-sample t-test was performed to probe the differences in the total and all five subdomain embodiment scores between the visuomotor synchrony IVR and asynchrony video experiences. We conducted a Pearson correlation analysis between the SoE scores and the total and all subdomain perceptions of re-experience rating scores to verify how participants re-experienced the visuomotor synchrony IVR and asynchrony video experiences within the MRI scan. Statistical analyses were performed using the Statistical Package for the Social Sciences (SPSS) software version 25 (IBM SPSS Inc. Chicago, IL, United States) for Windows, and statistical significance was set at *p* < 0.05.

#### 2.6.2 fMRI data analysis

We conducted preprocessing using statistical parametric mapping software (SPM12; Wellcome Department of Imaging Neuroscience, Institute of Neurology, London, United Kingdom) implemented in MATLAB R2017b (Mathoworks Inc., Natick, MA, United States). Preprocessing included correction for head motion, co-registration to anatomical images, spatial normalization using the anatomical image and the Montreal Neurological Institute template, and smoothing using a Gaussian kernel with a full width at half maximum of 6 mm. None of the registrations of the participants’ images differed between baseline and post-experience.

A conventional two-level fMRI analysis was performed using SPM12 (Friston et al., 1990; Friston et al., 1991). For the first-level analysis, the degree of activation or deactivation during the depression avatar auditory fMRI task was estimated for each participant using a general linear model (GLM). Preprocessed images of the baseline and post-experiences and two hemodynamic models (visuomotor synchrony IVR baseline > visuomotor synchrony IVR post-experience, visuomotor synchrony IVR baseline < visuomotor synchrony IVR post-experience, asynchrony video baseline > asynchrony video post-experience, and asynchrony video baseline < asynchrony video post-experience contrasts) were constructed using the standard hemodynamic function supplied by SPM12. Six estimated head-motion parameters were included in the GLMs as confounding factors.

In the second-level analysis, the neural correlates of individual differences in SoE scores were estimated using a voxel-by-voxel multiple regression analysis of the depression avatar auditory task responses across participants for visuomotor synchrony IVR and asynchrony video experiences. For each negative and positive depression avatar auditory task response, the total SoE scores for the visuomotor synchrony IVR and asynchronous video experiences were included as independent variables of interest. Age and sex were included as covariates in this analysis. Next, the effect of the depression avatar stigma auditory fMRI task response at the peak voxels of the identified activation clusters for the visuomotor synchrony IVR experience response was verified for the depression avatar stigma auditory fMRI task to the asynchrony control experience response and vice versa using a liberal statistical threshold for the region-of-interest (ROI) analysis. The ROI approach verified whether the activation peaks observed in the visuomotor synchrony IVR experience were also observed in the asynchrony video experience and vice versa. The voxel-wise statistics used an uncorrected *p* < 0.001 for the cluster-forming threshold, which was set at a family wise error-corrected *p* < 0.05 for cluster extent. The statistical threshold for the ROI analyses was set to an uncorrected *p* < 0.05. The identified brain structures were labeled using the SPM Anatomy toolbox (Eickhoff et al., 2005).

## 3 Results

Thirty-six healthy adults (13 female; age [mean ± *SD*] = 24.00 ± 6.1 years, age range = 19.0–46.0 years) fulfilled the inclusion criteria and were randomly assigned to the visuomotor synchrony IVR–asynchrony video group (visuomotor synchrony IVR Day 1 and asynchrony video Day 2 sequence; 18 participants) and the asynchrony video–visuomotor synchrony IVR group (asynchrony video Day 1 and visuomotor synchrony IVR Day 2 sequence; 18 participants). Of the 36 participants, two did not attend Day 2 of the visuomotor synchrony IVR experience, and two were excluded because of excessive head movements (i.e., > 7 mm) in the MRI scanner. Therefore, 32 participants completed all the assessments, experiences, and fMRI scanning: 18 participants (8 female; age [mean ± *SD*] = 23.87 ± 5.52 years) in the visuomotor synchrony IVR–asynchrony video group and 14 participants (5 female; age [mean ± *SD*] = 24.51 ± 7.41 years) in the asynchrony video–visuomotor synchrony IVR group. There were no statistical differences in demographic characteristics, such as age (*p* = 0.78) and sex (*p* = 0.62). Thus, we analyzed behavioral and brain data from 32 participants (13 female; age range = 19–46 years, age [mean ± *SD*] = 24.15 ± 6.21 years).

### 3.1 Behavioral data

#### 3.1.1 Comparison between visuomotor synchrony IVR and asynchrony video embodiment experiences

**Figure 3:**
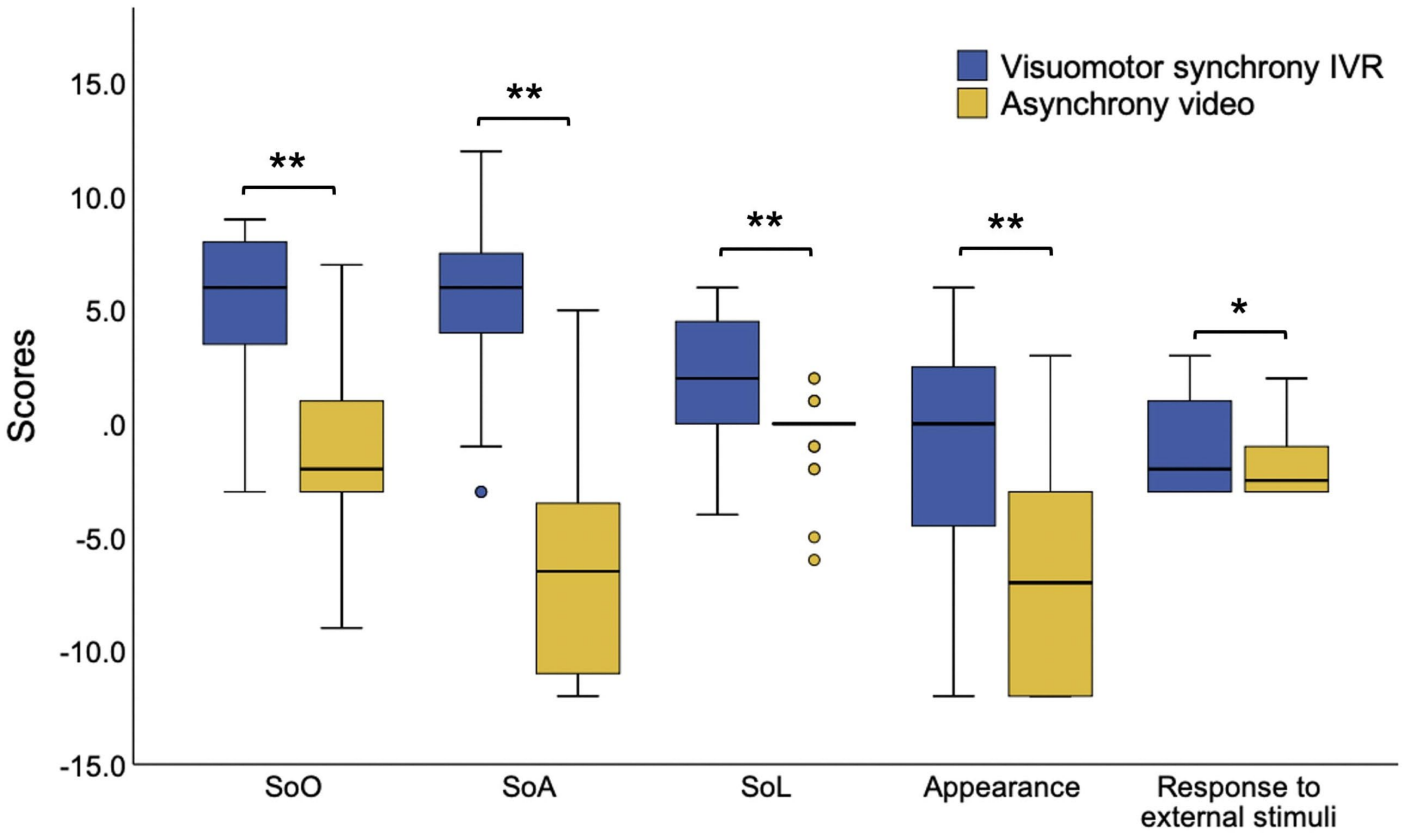
The SoE subdomains scores during the use of depression avatar on stigma context during the visuomotor synchrony IVR experience and asynchrony video experience. IVR, immersive virtual reality; SoE, sense of embodiment, SoO, sense of ownership, SoA, sense of agency, SoL, sense of localization, *p < 0.05, **p < 0.01.

The paired sample *t*-test revealed a significant high score of SoE (visuomotor synchrony IVR [mean ± *SD*] = 0.95 ± 0.87; asynchrony video [mean ± *SD*] = −0.99 ± 0.81, *t* (31) = 10.84, *p* < 0.01) and all five subdomains of SoE (SoO, visuomotor synchrony IVR [mean ± *SD*] = 5.25 ± 3.34, asynchrony video [mean ± *SD*] = −1.06 ± 3.24, *t* (31) = 7.83, *p* < 0.01; SoA, visuomotor synchrony IVR [mean ± *SD*] = 5.78 ± 3.91, asynchrony video [mean ± *SD*] = −6.63 ± 4.81, *t* (31) = 11.83, *p* < 0.01; SoL, visuomotor synchrony IVR [mean ± *SD*] = 2.41 ± 2.56, asynchrony video [mean ± *SD*] = −0.34 ± 1.60, *t* (31) = 5.57, appearance, visuomotor synchrony IVR [mean ± *SD*] = −0.97 ± 5.13, asynchrony video [mean ± *SD*] = −6.69 ± 4.98, *t* (31) = 7.36, *p* < 0.01; response to external stimuli, visuomotor synchrony IVR [mean ± *SD*] = −1.00 ± 1.92, asynchrony video [mean ± *SD*] = −1.84 ± 1.59, *t* (31) = 2.53, *p* < 0.05) during visuomotor synchrony IVR compared with the asynchrony video experience (Figure 3).

#### 3.1.2 Association between embodiment and perception of re-experience

Significant and marginally significant positive correlations were detected between the SoE score and the total perception of the re-experience score for visuomotor synchrony IVR (*r* = 0.52, *p* < 0.01) and asynchrony video (*r* = 0.35, *p* = 0.05) experiences, respectively; that is, participants with higher SoE scores for both types of experiences experienced high sensation during the fMRI experience. In terms of the subdomains of the perception of re-experience score for visuomotor synchrony IVR, the four perception subdomains were observed as weak to moderate, ranging from 0.40 to 0.48 (“sense of being there” *r* = 0.44; “sense of watching a movie” *r* = 0.41; “sense of personal experience” *r* = 0.40, *p* < 0.05; “sense of being a patient” *r* = 0.48, *p* < 0.01). A moderate correlation strength (“sense of family/friend” *r* = 0.41, *p* < 0.05) was observed for only one perception subdomain in the asynchronous video experience. The correlations between SoE and other perception subdomains and the re-experience score for the visuomotor synchrony IVR and asynchronous video experiences were not significant (Table 1).

**Table 1:** Correlation between SoE score during depression avatar experience with the perception of re-experiencing of the visuomotor synchrony IVR and asynchrony video experiences (*n* = 32).

	SoE during depression avatar experience			
	Visuomotor synchrony IVR		Asynchrony video	
	*r*	*p*-value	*r*	*p*-value
Perception of re-experience				
Negative words	0.22	0.22	0.08	0.68
Positive words	0.13	0.50	0.05	0.79
Visual and not words	0.16	0.40	0.12	0.52
Neither visual nor words	0.03	0.89	0.27	0.13
Sense of being there	0.44	0.01*	0.08	0.65
Sense of watching a movie	0.41	0.02*	0.27	0.14
Sense of being a patient	0.48	0.01**	0.26	0.15
Sense of depression	0.35	0.05	0.16	0.38
Sense of family/friend	0.16	0.38	0.41	0.02 *
Sense of personal experience	0.40	0.03*	0.18	0.32
Total score	0.52	0.01**	0.35	0.05^†^

Pearson’s correlation coefficients (r) between the SoE score and the perception of re-experiencing the depression avatar during visuomotor synchrony IVR and asynchrony video experience. SoE, sense of embodiment; IVR, immersive virtual reality, *p < 0.05, **p < 0.01, ^†^*p* = 0.05.

#### 3.1.3 The effect of visuomotor synchrony IVR and asynchrony video embodiment-related brain activities

**Figure 4:**
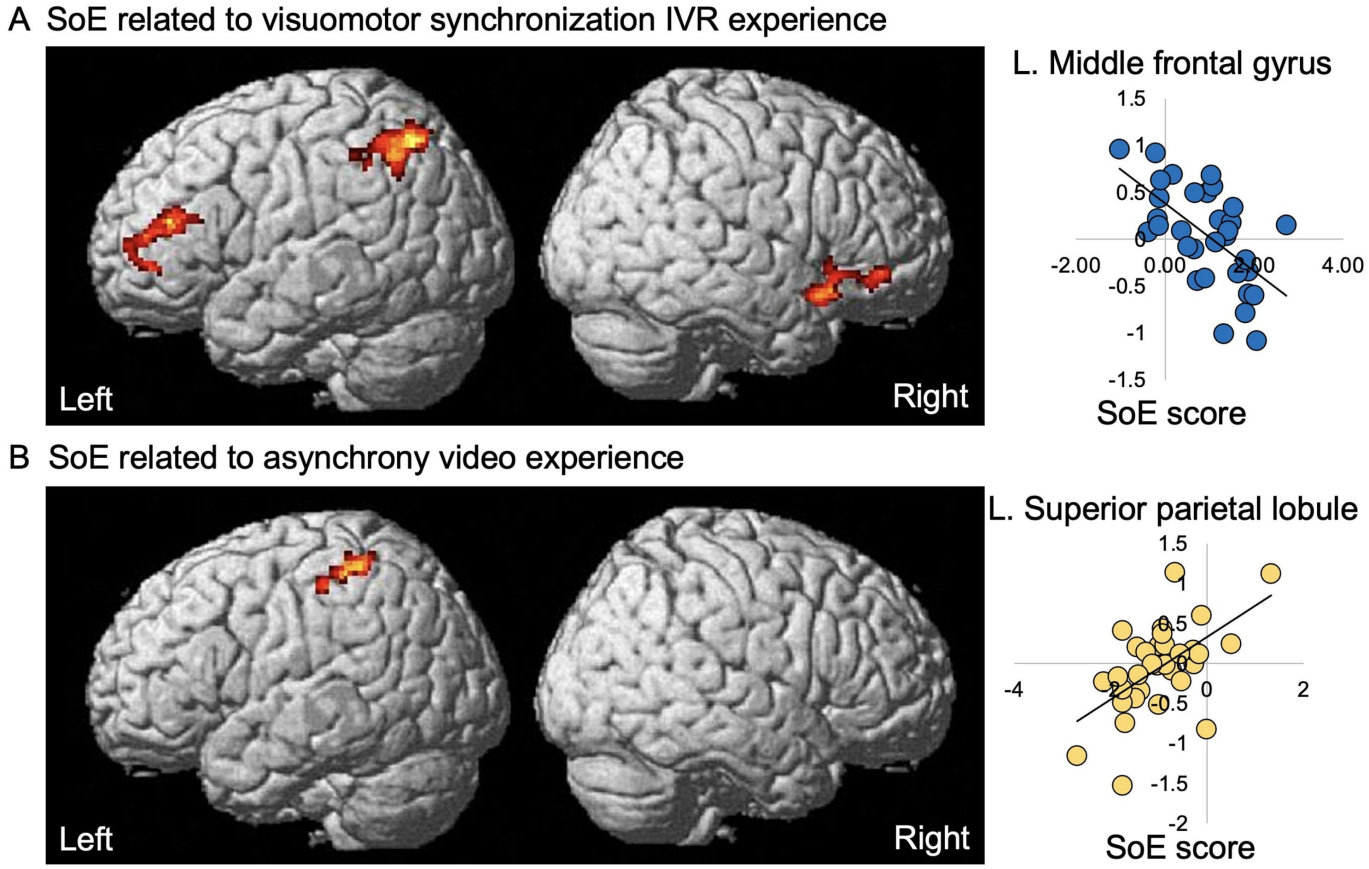
Significant effects of the SoE score on experience-related activation in a visuomotor synchrony IVR and asynchrony video of an avatar with depression experiencing stigma. Left panels, significant negative effect of the SoE response on visuomotor synchrony IVR experience-related activation **(A)** and significant positive effect of SoE response on asynchrony experience-related activation **(B)** of an avatar with depression experiencing stigma are superimposed on the standard SPM12 anatomical brain. Right panel, estimate (*β*) of visuomotor synchrony IVR and asynchrony experience-related activation (vertical axis) of each individual plotted against the significantly associated SoE score (horizontal axis); that is, the SoE response for the visuomotor synchrony IVR experience **(A)** and SoE response for the asynchrony video experience **(B)**; regression line is given for each plot. SoE, sense of embodiment; IVR, immersive virtual reality; R, right; L, left.

The results of voxel-wise regression analyses of the SoE score with stigma experience-related activation for both the visuomotor synchrony IVR and asynchrony experiences are summarized in Table 2 and Figure 4. With regard to visuomotor synchrony IVR post-experience (i.e., visuomotor synchrony IVR baseline < visuomotor synchrony IVR post), a significant negative effect of the SoE score was identified in the left MFG, right IFG, right AIN, left AnG, and left SPL. For the asynchronous video experience (i.e., asynchronous video baseline < asynchronous video post-experience), a significant positive effect of the SoE score was identified in the left supramarginal gyrus, left SPL, and left postcentral gyrus. No significant visuomotor synchrony IVR baseline activation (i.e., visuomotor synchrony IVR baseline > visuomotor synchrony IVR post) or asynchrony video baseline activation (i.e., asynchrony video baseline > asynchrony video post-experience) was identified.

**Table 2:** The significant effect of depression avatar on stigma experience for visuomotor synchrony IVR and asynchrony experiences in SoE score.

Brain region	Peak				Cluster		ROI	
	*x*	*y*	*z*	*t*	*k*	*p*	*t*	*p*
SoE during visuomotor synchrony IVR								
L. Middle frontal gyrus	−34	52	2	4.12	268	0.003	–	–
*R. Inferior* frontal gyrus	44	−40	−6	4.11	255	0.004	–	–
R. Anterior insula	42	24	−8	4.44			–	–
L. Angular gyrus	−46	−60	50	5.97	334	0.001	–	–
*L. Superior* parietal lobe	−24	−70	56	4.06			–	–
SoE before visuomotor synchrony IVR								
*n.s.*								
SoE during asynchrony								
L. Supramarginal gyrus	−46	−28	52	4.57	193	0.013	1.85	< 0.05
*L. Superior* parietal lobe	−34	−44	58	4.90			–	–
L. Postcentral gyrus	−40	−36	56	4.27			–	–
SoE before asynchrony								
*n.s.*								

The coordinates (x, y, z) and the *t*-value of peak activation, the size and p-value of the clusters are given for baseline < post experience and baseline > post experience in the depression avatar on stigma experience for visuomotor synchrony IVR and asynchrony embodiment-related brain activation (i.e., regression analysis). IVR, immersive virtual reality; SoE, sense of embodiment; ROI, region of interest; L, left; R, right; n.s., not significant.

## 4 Discussion

This study investigated how humans acquire virtual body representation through SoE subcomponents and the neural correlates of individual differences in SoE using the visuomotor synchrony IVR of an avatar with depression experiencing stigma. Participants underwent the visuomotor synchronization IVR of a depression avatar in a stigma context experience (e.g., to receive the document that was rejected by the boss by grabbing a paper) and watched the same content on a conventional 2D monitor (asynchronous video experience). The asynchronous video experience content was the same as the visuomotor synchrony IVR experience, but without immersion and visuomotor synchronization. We found that the visuomotor synchrony IVR experience showed higher scores on all five SoE subcomponents, including the SoE, compared to the asynchronous video experience. Furthermore, we found decreased brain activity in the left MFG, right IFG, right AIN, and left PPC while developing the SoE in the visuomotor synchrony IVR experience, and increased brain activity only in the left PPC while developing the SoE in the asynchronous video experience. As discussed below, acquiring the SoE using visuomotor synchrony IVR differs from the asynchrony video experience in terms of capacity to elicit different types of perceptions where the self is placed in the position of the depression avatar (i.e., “sense of being a patient,” “sense of personal experience”) and the visuomotor synchrony IVR embodiment-related brain activations.

Gradual visuomotor synchronized movements of a part of the depression avatar’s body (i.e., right hand) were sufficient to generate significantly high scores in all five SoE subdomains. Previously, we showed a decrease in the experience of stigma in a person with mild depression (Lem et al., 2024). Our findings are in accordance with a recent study that showed that high SoO can improve identification with female victims of domestic violence through head-synchronized visuomotor movements (de Borst et al., 2020). In contrast, many studies (Kilteni et al., 2013; Peck et al., 2013; Banakou et al., 2016; Banakou et al., 2018) have used full body visuomotor synchronization embodiment with a stigmatized avatar to obtain positive changes in the perception, attitude, and behavior toward the stigmatized group. Thus, our visuomotor synchronization IVR experience of an avatar with depression was able to acquire a body representation through the contribution of all five SoE subcomponents using a part of the body (i.e., the right hand) of the avatar with depression.

### 4.1 Decreased brain activity in the frontoparietal and AIN reflects embodiment of an avatar with depression in a visuomotor synchrony IVR experience

The neural findings on the SoE score for visuomotor synchrony with the avatar with depression IVR experience agrees with the view that frontoparietal activity reflects an individual’s tendency to feel embodiment with the depression avatar and perceive the depression avatar as one’s own body through different sensory information and personal perceptive experiences. In terms of sensory information, the visuomotor synchronization IVR experience used multisensory information (i.e., vision, motor function, proprioception, hand location, and appearance), which was indicated by the high scores in the five subdomains of the SoE. Interestingly, unlike the asynchronous video experience, which was related to only one subdomain and also distant personal perception (i.e., “sense of family/friend”), the SoE in the IVR was correlated with different types of personal perception experiences (i.e., “sense of being a patient”). According to previous fMRI studies, the frontal brain regions (i.e., the left MFG and IFG) were related during periods when participants experienced the induction of the RHI and body swap illusion (Petkova et al., 2011; Guterstam et al., 2013; Serino et al., 2013; Chae et al., 2015; Grivaz et al., 2017). Furthermore, activity in the PPC brain regions (i.e., the left SPL, AnG, and IPS) is related to multisensory integration during visuo-tactile synchronization (Petkova et al., 2011; Guterstam et al., 2013; Grivaz et al., 2017). In terms of individual variation in visuomotor synchronization, the greater the sense of presence the participants had toward the 3D videos of the VR environment, the lower their neural activation in the MFG (Clemente et al., 2014). For the IFG and AnG, the SoA for controlling an avatar were negatively associated with increased activity in the IFG and AnG (Cai et al., 2024). Thus, the function of illusion induction generated by multisensory integration in the frontoparietal region is related to the transformation of visual, motor, proprioceptive, hand location, and appearance signals into a stable spatial representation of the hand of the avatar with depression, which uses different sensory and perceptual modalities.

We also observed a visuomotor synchrony IVR embodiment-related decrease in brain activity in the right AIN, which suggests that the SoE induces changes in the interoceptive process. Previous studies have revealed the involvement of the AIN in the RHI (Guterstam et al., 2013; Limanowski et al., 2014; Chae et al., 2015) and body swap illusion (Petkova et al., 2011; Serino et al., 2013) through visuo-tactile synchronization. In addition, the AIN is activated when participants experience fear responses during SoE visuomotor synchronization related to the approach of a threatening virtual avatar in 3D VR videos or a male virtual avatar who verbally assaults and throws a telephone to the floor in an IVR environment (Seinfeld et al., 2021; de Borst and de Gelder, 2022). Furthermore, decreased activation of the AIN is associated with embodiment in victims of virtual abuse (Seinfeld et al., 2021). Thus, we speculate that the right AIN activity reflects changes in the interoceptive process associated with the emotional responses provoked by the visuomotor synchronization of the IVR depression avatar embodiment in the context of stigma.

### 4.2 Increased brain activity in the PPC in the asynchrony video embodiment experience

In our asynchrony video experience, we excluded motor stimuli while keeping the visual input constant and obtained a low SoE score compared to the visuomotor synchrony IVR SoE experience. In addition, the bilateral frontal and right AIN activation disappeared with increasing PPC activation. Our results extend previous fMRI studies on the RHI by demonstrating that the activation of these areas is important for multisensory integration function in the PPC, which was previously related to the transformation of visual, tactile, and proprioceptive signals into a stable spatial representation of body parts that use different sensory modalities (Serino et al., 2013).

The strength of this study lies in its novelty, as no previous study has demonstrated the relationship between frontoparietal and AIN regions with the individual tendency to feel embodied with an avatar with depression and perceive the avatar with a senses of one’s own body through different sensory information and perceptive personal experiences using a combination of IVR and fMRI. However, our study has some potential limitations. First, although we could monitor instantaneous changes in the brain with high spatial resolution and specific effects during the relief of the visuomotor synchronization IVR experience, it was not possible to monitor the visuomotor synchronization IVR experience directly because of the limitations of the magnetic materials and movement of the MRI scanner. In other words, the participants were instructed to visualize the visuomotor synchronization movements and listen to an audio recording of the IVR experience during the fMRI scan, rather than physically engaging in the full IVR experience. To partially overcome these limitations, we asked the participants to report how they relied on imagining the SoE experience within the MRI scanner. The participants reported no problems related to both types of experiences with the MRI scanner, which can be observed through the significant and marginally significant positive correlations between SoE and the total perception of re-experience in the MRI for visuomotor synchrony IVR and asynchrony video experience, respectively. Furthermore, imagination can elicit real-time physiological and emotional responses similar to those occurring in real situations (Ehrsson et al., 2003; Christian et al., 2015). Second, we should have investigated whether a single-day IVR session involving a virtual avatar with depression can induce a state sufficiently analogous to mild depression. Although, in terms of behavior, our previous study (Lem et al., 2024) showed that there was a significantly higher score in the sense of depression and sense of being a patient for a single-day of the virtual avatar with depression session compared to single-day video session. Last, this study did not explore others potential individual differences (e.g., personality traits, prior experience with virtual reality, mental health status), and participants’ psychological state or empathy toward the avatar that could influence the degree of embodiment experienced.

## 5 Conclusion

Our data deepen our understanding of the neural mechanisms underlying individual differences in the SoE. Here, we demonstrated that the visuomotor synchronization IVR-based embodiment of the hand of an avatar with depression elicited changes in all five SoE subcomponents, as well as changes in frontoparietal and AIN brain activity. The novel fMRI-IVR approach adopted here may expand our knowledge of the neural basis of SoE and may be useful for developing new IVR-based intervention approaches.

## Data Availability

The datasets presented in this article are not readily available because due to the risk of identifying the participants from reconstructed images. However, the behavioral data are available from the corresponding author (KHdSK) upon reasonable request, including a project outline. Requests to access the datasets should be directed to Kelssy Hitomi dos Santos Kawata, kawatakelssy@gmail.com.
